# Discovery and disentanglement of aligned residue associations from aligned pattern clusters to reveal subgroup characteristics

**DOI:** 10.1186/s12920-018-0417-z

**Published:** 2018-11-20

**Authors:** Pei-Yuan Zhou, Antonio Sze-To, Andrew K. C. Wong

**Affiliations:** Systems Design Engineering, University of Waterloo, Waterloo, ON Canada

**Keywords:** Pattern discovery, Disentanglement, Aligned residue associations, Aligned pattern clusters, Subgroup characteristics

## Abstract

**Background:**

A protein family has similar and diverse functions locally conserved. An aligned pattern cluster (APC) can reflect the conserved functionality. Discovering aligned residue associations (ARAs) in APCs can reveal subtle inner working characteristics of conserved regions of protein families. However, ARAs corresponding to different functionalities/subgroups/classes could be entangled because of subtle multiple entwined factors.

**Methods:**

To discover and disentangle patterns from mixed-mode datasets, such as APCs when the residues are replaced by their fundamental biochemical properties list, this paper presents a novel method, Extended Aligned Residual Association Discovery and Disentanglement (E-ARADD). E-ARADD discretizes the numerical dataset to transform the mixed-mode dataset into an event-value dataset, constructs an ARA Frequency Matrix and then converts it into an adjusted Statistical Residual (SR) Vector Space (SRV) capturing statistical deviation from randomness. By applying Principal Component (PC) Decomposition on SRV, PCs ranked by their variance are obtained. Finally, the disentangled ARAs are discovered when the projections on a PC is re-projected to a vector space with the same basis vectors of SRV.

**Results:**

Experiments on synthetic, cytochrome c and class A scavenger data have shown that E-ARADD can a) disentangle the entwined ARAs in APCs (with residues or biochemical properties), b) reveal subtle AR clusters relating to classes, subtle subgroups or specific functionalities.

**Conclusions:**

E-ARADD can discover and disentangle ARs and ARAs entangled in functionality and location of protein families to reveal functional subgroups and subgroup characteristics of biological conserved regions. Experimental results on synthetic data provides the proof-of-concept validation on the successful disentanglement that reveals class-associated ARAs with or without class labels as input. Experiments on cytochrome c data proved the efficacy of E-ARADD in handing both types of residue data. Our novel methodology is not only able to discover and disentangle ARs and ARAs in specific statistical/functional (PCs and RSRVs) spaces, but also their locations in the protein family functional domains. The success of E-ARADD shows its great potential to proteomic research, drug discovery and precision and personalized genetic medicine.

## Background

**Fig. 1:**
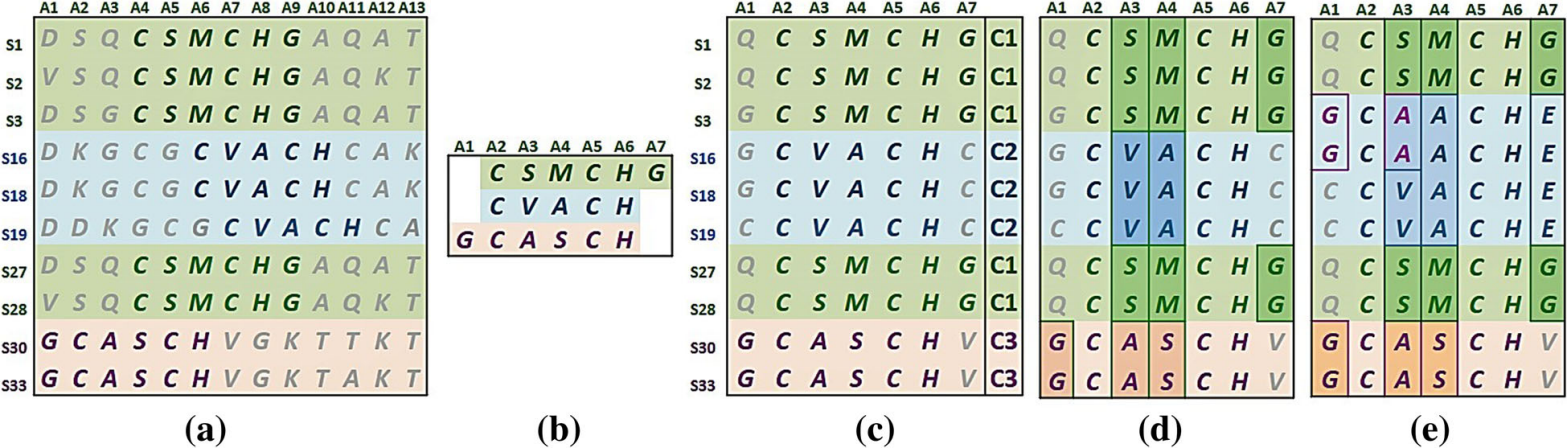
Pattern and Data Spaces of APC and ARAs. **a** A portion of protein sequence dataset with discovered high order patterns (in bold) [2] with labels on the top row denote the aligned sites, on the first column denote the sequence ID; (**b**) Aligned Pattern Cluster (APC) Pattern Space obtained [3]. **c** APC Data Space (APC-D). C1, C2, C3 represents three classes. **d** An discovered ARA Cluster contains three partitioned subgroups and displayed in green, blue and red shade associate with class C1, C2 and C3 respectively. **e** Entangled ARAs. For example, in S3 S16, the AVAs A1G and A3A in C2 are from C3 entangling with its ARAs A4A and A7E

Proteins and their interactions control the biological process of a living organism. Within the same family, proteins have similar functions. Thus, discovering conserved sequence patterns from a family is crucial for revealing domain functionality. However, due to mutations and/or multiple functionality, even these conserved patterns may have substantial differences in species or even functions. Hence, identifying subgroup characteristics are of fundamental importance. We have developed a novel method to obtain knowledge-rich [1] Aligned Pattern Clusters (APC) [2–4] from protein families (Fig. 1(a) and (b)) to represent biological conserved regions. Figures 1(b) and (c) show its pattern space (APC) and data space (APC-D) respectively [2–4]. When a local functional domain is identified, and class labels are given, it is easy to see how the ARs and ARAs are entangled among different classes within the conserved domain (Fig. 1(c)) if the data size is small. We may be able to disentangle their class relation. However, if the size of data is large and more subtle classes or subgroups are present while the class labels are unknown (Fig. 1(d)), the task of ARA disentanglement becomes extreme difficult. To overcome this challenge, a novel algorithm denoted as Aligned Residue Association Discovery and Disentanglement (ARADD) [5], has been developed by us, where ARADD is originated from our recent best-paper-award work [6], by considering the aligned sites in an APC as attributes and residues on a site as attribute values. Hence, we extend AVADD to E-ARADD (Aligned Residue Association Discovery and Disentanglement) to obtain succinct disentangled subgroups of ARAs, revealing more succinct stereo physiochemical knowledge of the conserved regions with or without explicit reliance of class labels. Since this knowledge is not obvious in the data, we refer it as deep knowledge discovered.

It should be noted that to discover knowledge at the physiochemical level, we have to handle mixed-mode data, i.e. data containing both categorical and numerical values. This becomes an interesting challenge since physiochemical properties in Aligned Pattern Clusters [2–4], apart from our early work [7] have not yet been seriously explored. In the following paragraphs, we provided a brief introduction of the related work, ranging from association rule mining to pattern discovery in protein sequences.

### Pattern discovery and association rule mining

In the field of data mining, association rule mining [8] is common to mine itemset from relational tables. Algorithms such as Apriori [9] and FP-growth [10] are used to capture associations from relational dataset. However, the above algorithms are extremely sensitive to parameters and thresholds setting, such as probabilistic thresholds, the number of clusters, distance measure and so on. Furthermore, a challenging problem encountered that the discovered patterns may be masked or obscure in the data due to the entanglement of unknown factors in their source environment [5, 6]. Therefore, for the real-world applications in Bioinformatics with noise in the data, it is important to discover patterns in a robust manner to enhance biological comprehension and interpretation.

### Protein functional regions represented by aligned pattern clusters

Protein sequence analysis is crucial for identifying and understanding the functional regions, as protein structures are expansive to obtain. Multiple Sequence Alignment (MSA) and Motif Discovery are the two major methods. Given an entire set of protein sequences, MSA [11–13] aligns them globally to identify the conserved regions. However, MSA is limited as it is only suitable for globally homologous sequences with a high level of sequence similarity [13]. Different from MSA, Motif Discovery [14, 15] locates and aligns similar subsequences locally to construct a probabilistic model for representing the aligned amino acids. However, motif discovery makes unrealistic assumption that there is independence between residue columns to represent the conserved sequence patterns, where in reality it is clearly not the case [16, 17]. Aligned Pattern Cluster (APC) [2–4] was thus developed to discover sequence patterns directly, and to capture functional conserved residue association in order to identify clusters of aligned patterns from the sequence data. Since APCs conserve both strong statistical sequence associations and homologous sites, it is more knowledge rich [2, 3] to reveal similar yet diverse functional associations in protein families.

### Physiochemical properties in aligned pattern clusters

In this study, we extend ARADD [5] to E-ARADD to discover physiochemical subgroup patterns in APCs at the residue (amino acid) level and the deeper level with mixed-mode residue physiochemical property. Hence, the ARPA clusters discovered can directly reveal the physiochemical characteristics of the APCs. We refers them as APPC patterns. In the notations, we insert term “Property” by adding the character “P” into AR, ARA and APC as ARP, ARPA and APPC respectively while the theory and the algorithm are not affected. We thus use them interchangeably except in some specific situation.

### Novelty and contributions

The novelty of this study, is the consolidation of our recent work [5] and the extension of our ARADD algorithm [5] into E-ARADD. We introduced into E-ARADD the Aligned Residue Property (ARP), an ordered tuple for five biochemical properties for Aligned Residue Property Association (ARPA) Pattern Discovery and Disentanglement. Additional experimental analyses were conducted to support our proposed algorithms. Besides, we used the Adjusted Statistical Residual instead of standard statistical residual to measure the significance of discovered associations so as to give a more accurate indication of how far the observed count deviates from the expected count to evaluate the statistical significance of ARA/ARPA.

The major contributions of our study are three-folded.

1. We extended the previous ARADD into E-ARADD to handle the mixed-mode physiochemical protein data with chemical properties for direct residue biochemical association interpretation.
2. We showed that sequence patterns could be discovered and disentangled from APCs, even if the patterns were mixed or entangled in functionality and location.
3. We validated that E-ARADD could reveal functional subgroups and subgroup characteristics of APCs and locate their residing domains through the case study on Class A Scavenger Receptor family (SR-A). Understanding subgroup characteristics of conserved regions in proteins could render new knowledge for gene therapy applications [18].

## Methods

**Fig. 2:**
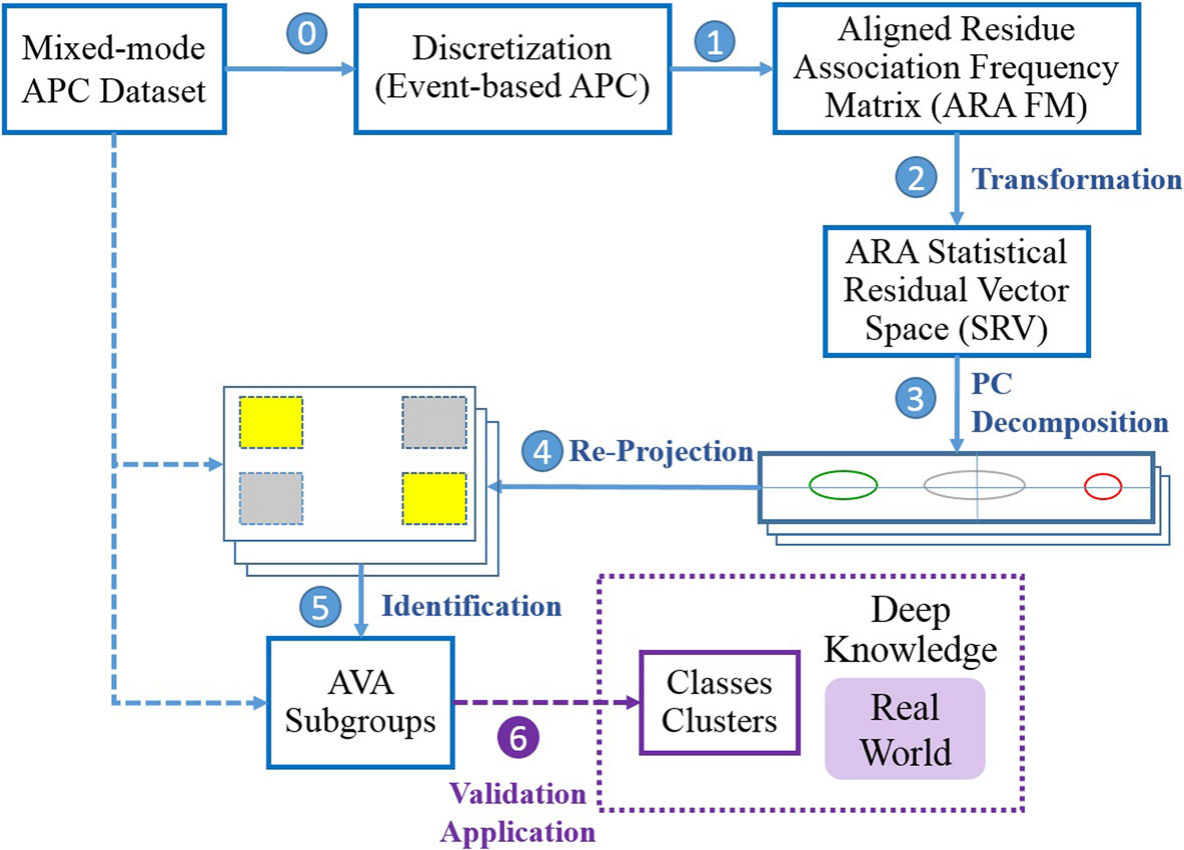
Schematic Overview of E-ARADD

This study focuses on discovering inherent ARAs/ARPA from APCs; clustering them into subgroups to reveal the functionalities of proteins within conserved functional regions and discover deep knowledge (PC/RSRVs) from APCs. Table 1 gives an abbreviation of terms and Fig. 2 provides a schematic overview of our method.

**Table 1:** Notations and terminologies

APC	Aligned Pattern Cluster (with categorical amino acid symbols)
APPC	Aligned Property Pattern Cluster (with mixed-mode chemical properties)
AR	Aligned Residue (for amino acid symbols in APC dataset)
ARP	Aligned Residue Property (for mixed-mode chemical properties in APC dataset)
ARA	Aligned Residue Association (Significant co-occurrence of two ARs in APCs)
ARPA	Aligned Residue Property Association (for APPC)
ARA/ARPA FM	ARA/ARPA Frequency Matrix
SR	adjusted Statistical Residual between two ARs/ARPs
SRV	ARA/ARPA adjusted Statistical Residual Vector Space
PCD	Principle Component Decomposition
RSRV	Re-projected SRV

**Fig. 3:**
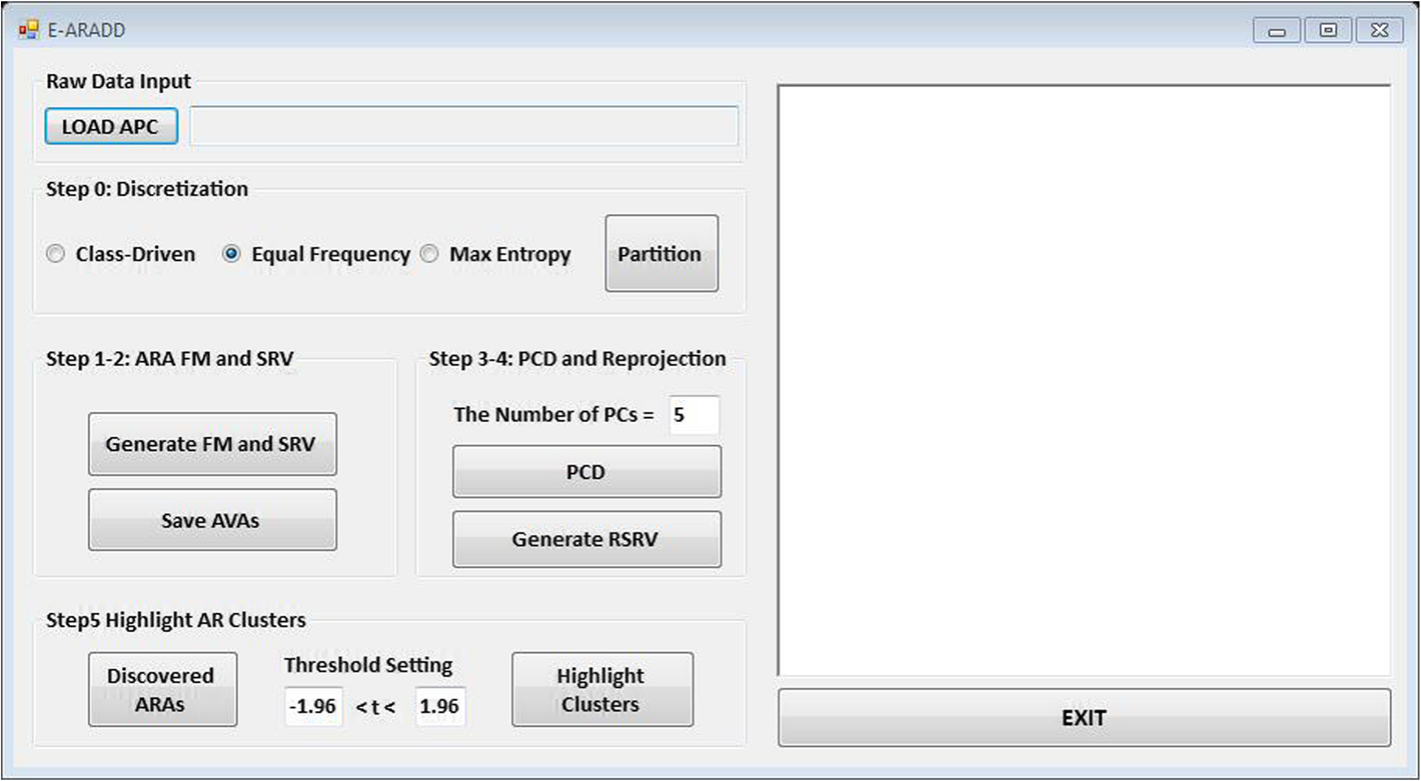
E-ARADD prototype of Interactive Decision Support GUI

To show that ARADD can go one level deeper to discover and disentangle ARA at the aligned residue chemical property level, we replace each aligned residue in an APC by its five-tuples of chemical properties referred to as APPC. Given a mixed-mode APC dataset, E-ARADD can accomplish the followings in steps as circled in Fig. 2. In addition, Fig. 3 shows how E-ARADD could be easily shifted from operating modes of APC and APPC via an Interactive GUI to visualize the use of the proposed algorithm.

In the most general setting, an APC/APPC is represented by **R**. Every tuple in **R,** denoted as A = {*A*_1_, *A*_2_, …*A*_*N*_}, is described by N amino acid sites or the five chemical properties of the residues (ARP tuples) in the aligned sites.

First, to discover event (residue property) associations, the numerical values of the source data need to be discretized into intervals. Discretization can minimize the impact of noisy data in the data mining process [19]. It also can help smooth data to reduce noise [20], speed up classification process [21] and make classification result more meaningful and easier-to-understand [22]. Hence, as Fig. 2 shows, in step 0, a mixed-mode APC is first converted to a categorical APC by discretizing all the numerical (ordinal) chemical properties of amino acid into intervals.

Equal Width and Equal Frequency are two simplest discretization methods. However, if uncharacteristic extreme values (outliers) exist in the data set, *Equal Width* can hardly handle this situation [22]. Hence, we transform numerical chemical properties of amino acid into discrete value using *Equal Frequency* [22] algorithm. Besides, we also implemented two other algorithms, class-driven discretization [23], called Optimal Class-Dependent Discretization OCDD, when class labels are given, and equal probability maximizing the entropy [24] when class labels are not given. As Fig. 3 shows, the original mixed-mode APC can be transformed into a categorical one after selecting a discretization method and pushing the button labeled “Partition”.

Therefore, each amino acid site or chemical property *A*_*n*_ can assume a numerical value or a categorical value.

1. For a continuous value, *A*_*n*_ is partitioned into *I*_*n*_ bins by transforming the original numerical values of *A*_*n*_ into interval event values, denoted as ${A}_n=\left\{{A}_n^i|i=1,2,\dots {I}_n\right\}$. If the distinct value of numerical *A*_*n*_ is less than three, we treat it as a categorical attribute.
2. For categorical attribute, *A*_*n*′_ contains *I*_*n*′_ values, we denote it as ${A}_n=\left\{{A}_n^j|j=1,2,\dots {I}_{n\prime}\right\}$.

After transforming the mixed-mode dataset into an event-value dataset, all the values of an attribute (*A*_*n*_) can be denoted as ${A}_n=\left\{{A}_n^1,{A}_n^2,\dots {A}_n^{I_n}\right\}$.

Then, we will present the methodology through the algorithmic process with formal definitions and theoretical content as below

**Fig. 4:**
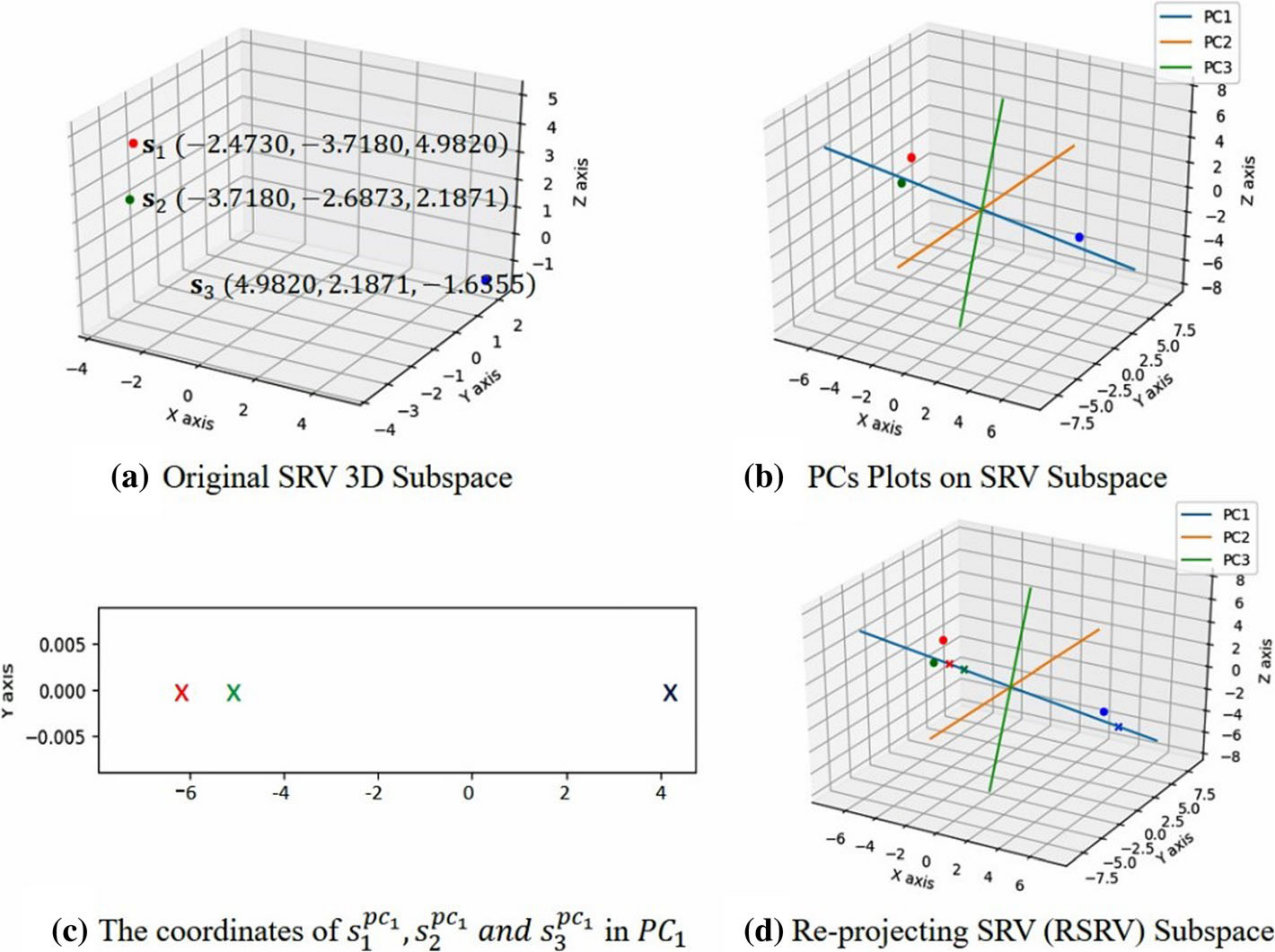
Diagrammatic Illustration of SRV, PC Plots and Their Coordinates in SRV and RSRV Subspaces. **a** Original data space: three a-vectors from the experiment is display in the 3-dimensional SRV Subspace. **b** PCs Plots: a-vectors position after applying PCD on SRV. **c** The coordinates of ${s}_1^{pc_1},{s}_2^{pc_1}\  and\ {s}_3^{pc_1}$ in *pc*_1_: the projection of the transformed a-vectors on the PC (as colored crosses). **d** Re-projecting the Coordinates in PCs: Re-projection of a-vector projections on the PC to RSRV subspace (as crosses on the blue axis). The colored dots are their original position in the SRV subspace

1. **Construct ARAFM** In step 1 (Fig. 2) we scan through the APC/APPC to construct an ARAFM/ARPAFM which is obtained from the frequency counts between two ARs/ARPs, say *FM(*${A}_n^i\leftrightarrow {A}_{n\prime}^j$*)*, where ${A}_n^i$ denotes the *i*^*th*^ value on the *n*^*th*^ aligned site/property in the APC/APPC, and ${A}_n^j$ denotes the *j*^*th*^ value on the *n*^′*th*^ aligned site/property in the APC/APPC (n≠*n*^′^). Hence ARAFM/ARPAFM is a *I* × *I* matrix, where *I =*$\sum \limits_{n=1}^N{I}_n$ represents the total number of event values of all sites in an APC/APPC.
2. **Obtain SRV.** In order to disentangle the statistical residuals by Principal Component Decomposition (PCD) [25], we first convert the ARAFM/ARPAFM into an adjusted statistical residual (SR) matrix, referred to as a SR Space (SRV), (Step 2 in Fig. 2) by converting each ARA/ARPA frequency in the ARAFM/ARPAFM into an adjusted SR value to account for the deviation of the observed frequency against the expected frequency if that ARA/ARPA is a random happening.
  Formally, ARAFM/ARPAFM is transformed into SRV by converting each ARA frequency into an SR, denoted as *SR(*${A}_n^i\leftrightarrow {A}_{n\prime}^j$*)* = *SR*_*ij*_ = $\frac{o_{ij}-{e}_{ij}}{\sqrt{e_{ij}}}$ . *o*_*ij*_ represents the total number of occurrence when *A*_*n*_= ${A}_n^i$ and ${A}_{n\prime }={A}_{n\prime}^j$; *e*_*ij*_ represents the expected value of *o*_*ij*_. *SR*_*ij*_ measures whether *o*_*ij*_ is significantly deviating from *e*_*ij*_ to reveal the statistical significance of an ARA/ARPA. At the confidence level of 95%, the discovered ARA/ARPA is positive significant or negative significant when its SR > 1.96 or SR < − 1.96; and if the SR is between − 1.96 and 1.96, the ARA/ARPA is considered as irrelevant or random occurrence.
  In order to disentangle the statistics in the SR matrix, we treat it as a vector space, denoted as SRV, where each row represents a vector corresponding to an AR (referred to as an AR-vector or just an *a*-vector) whose coordinates are the SRs of that AR associating with other distinct ARs (of other attributes) represented by the column *a*-vectors. Then, SRV can be represented as a set of vectors, denoted as, SRV = <${SRV}_{A_1^1},\dots {SRV}_{A_1^{I_1}},\dots {SRV}_{A_n^{I_n}},\dots {SRV}_{A_N^{I_N}}$>, where ${SRV}_{A_n^i}$={*SR(*${A}_n^i\leftrightarrow {A}_1^1$*), … SR(*${A}_n^i\leftrightarrow {A}_1^{I_1}$*),… SR(*${A}_n^i\leftrightarrow {A}_N^{I_N}$*)*} and *SR(*${A}_n^i\leftrightarrow {A}_n^i$*) = 0*.
3. **Disentangle the SRV by PCD**. In Step 3, we conduct PCD to disentangle the SRV into PCs ranked according to the descending order of their eigenvalues. In PCD, PCs are a sets *k* PCs, denoted as PC = {*PC*_1_, *PC*_2_, …*PC*_*k*_}, where *PC*_*n*_ is a set of projections of the a-vectors from SRV on it and denoted as *PC*_*n*_={${PC}_n\left({A}_n^i\right)\mid n=1,2,\dots N,i=1,\dots {I}_n$}, where N represents the total number of all ARs/ARPs and *I*_*n*_ represents the total number of distinct values of *A*_*n*_.Fig. 4 (a) to (c) give a diagrammatic illustration of applying PCD to the SRV. Considering a matrix, A (i.e. a three-dimensional subspace of SRV) with 3 points as shown in Fig. 4(a) of the original data space. After applying PCD, we obtain eigenvectors and eigenvalues, sorted in descending order according to the magnitude of their eigenvalues. Fig. 4(b) shows the PC axis induced by their projection of the a-vectors that maximize their variance on that PC. Fig. 4(c) shows the coordinates of the projection of the a-vectors on the PC.
4. **Re-project the** ***a*-vector projections on each PC**. In step 4, we re-project the projections of the a-vectors on the PC back to an SRV with the same basis vectors of the previous SRV. We refer this new SRV as the Re-projected SRV (denoted as RSRV) with subscript *k* in *RSRV*_*k*_ corresponding to that in *PC*_*k*_. *RSRV*_*k*_ is the SRV containing the transformed positions of a-vector on *PC*_*k*_ via *RSRV*_*k*_ = *SRV* ∙ *PC*_*k*_ ∙ *PC*_*k*_^*T*^.
  Figure 4(d) shows the new positions of the a-vectors representing their projection on the PC to the RSRV. In each RSRV, like SRV, each row represents an *a*-vector corresponding to an AR with a new set of coordinates accounting the statistical strength SRs of that AR associating with other ARs captured by the PC governed by certain specific underlying factors.
5. **Identify ARAs/ARPAs and AR/ARP Clusters in each PC.** Since each row a-vector in SRV represents an AR or their properties associating with other ARs or properties as its coordinates, the PC transformation will bring out in the PC the highest variance of the a-vectors with high SR coordinate values and display them at the far ends from the center (with zero coordinate value) of the PC. We may not see the reason why an a-vector is significant at the surface, but when viewing it in the RSRV, we would find out that the coordinate(s) of an a-vector of an AR/ARP reflect the statistic strength of its ARAs/ARPAs with another AR(s)/ARP(s) contributing to its high variance on the PC. In general, PCD is sensitive to the relative scaling of the original variables, often masking their distinctiveness. However, by converting the AR(P)AFM into SRV with uniform SR scale and statistical weights, both ARADD and E-ARADD utilize the statistical strength and functional decomposition to reveal more stable, subtle yet significant statistical associations that might be masked in the original frequency space. Hence, in this step, the significant AR(P)As discovered and disentangled are more distinct, stable and specific as manifested in separate RSRVs. Therefore, a cluster of ARs can be generated by AR(P)s that share strong AR(P)As.
  As Fig. 3 shows the GUI of E-ARADD server, for step 1–2, when pressing the button labeled “Generated FM and SRV” on E-ARADD server, both ARAFM and SRV are constructed for original data. Then, for step 3–4, the set of top PCs and their corresponding RSRVs are generated depending on the values of parameters (i.e. the number of PCs) are assigned in the box. Finally, in step 5, the sub-cluster results are highlighted according to the assigned confidence interval in the box.

Finally, we can validate the output RSRVs and AR(P) clusters when apply E-ARADD in specific application. We summarize the results as below.

1. The significant disentangled AR(P)As. These can be found from the distinct AR(P)s and the AR(P) clusters in the PCs based on their distance from the center (with zero value) of the PCs. When AR(P)As were entangled, the SRV disentanglement to reveal distinct AR(P)s in the PCs is crucial for yielding highly distinct, stable, and specific results as manifested in the RSRVs obtained from both datasets.
2. AR(P)s Sub-clusters. On one hand, the disentangled PCs can reveal significant AR(P)s/AR(P)-Clusters on a one-dimensional space; on the other hand, the SR of the AR(P)As in RSRVs can further reveal the significance of the AR(P)As and the AR(P)-Clusters. The AR(P) subgroups that are obtained in different orthogonal PC spaces may have functional meaning leading to established or new biological interpretation.

## Results

In this study, we conducted both experiments on synthetic data and bio-sequence data. We hereby illustrate the experimental results and their analysis in this section.

### Synthetic dataset

We first generated a 300 × 6 matrix with the first column representing class labels and the following 5 representing 5 attributes values AVs (equivalent to aligned residues ARs). First for each entry of an attribute column, we stochastically generated characters from a uniform distribution of the characters via a pseudo random number generator. We then embedded patterns of three different classes 1, 2 and 3 (*C*_1_, *C*_2_, and *C*_3_) as shown in Table 2. To simplify the notation, from here on we represent an attribute (say A1) assuming a certain value (say A) by A1A.

**Table 2:** Synthetic dataset with embedded entangled patterns

Classes	Attribute Values are Significant Associated with Class Label
C1	A1A, A2C, A3E, (A4 H/G, A5M/N) where A4 and A5 are random patterns
C2	A1A, A2D, A3F, (A4 H/G, A5M/N) where A4 and A5 are random patterns
C3	A1B, A2D, A3E, (A4 H, A5M/N) where A4 and A5 are random patterns

In Table 2, we can find that the attribute values A1A, A4H, A5N are entangled for class 1 and class 2; attribute values A3E, A4H, A5M and A5N are entangled in class 1 and class 3; and attribute values A2D, A4H, A5M, A5N are entangled in class 2 and class 3.

**Fig. 5:**
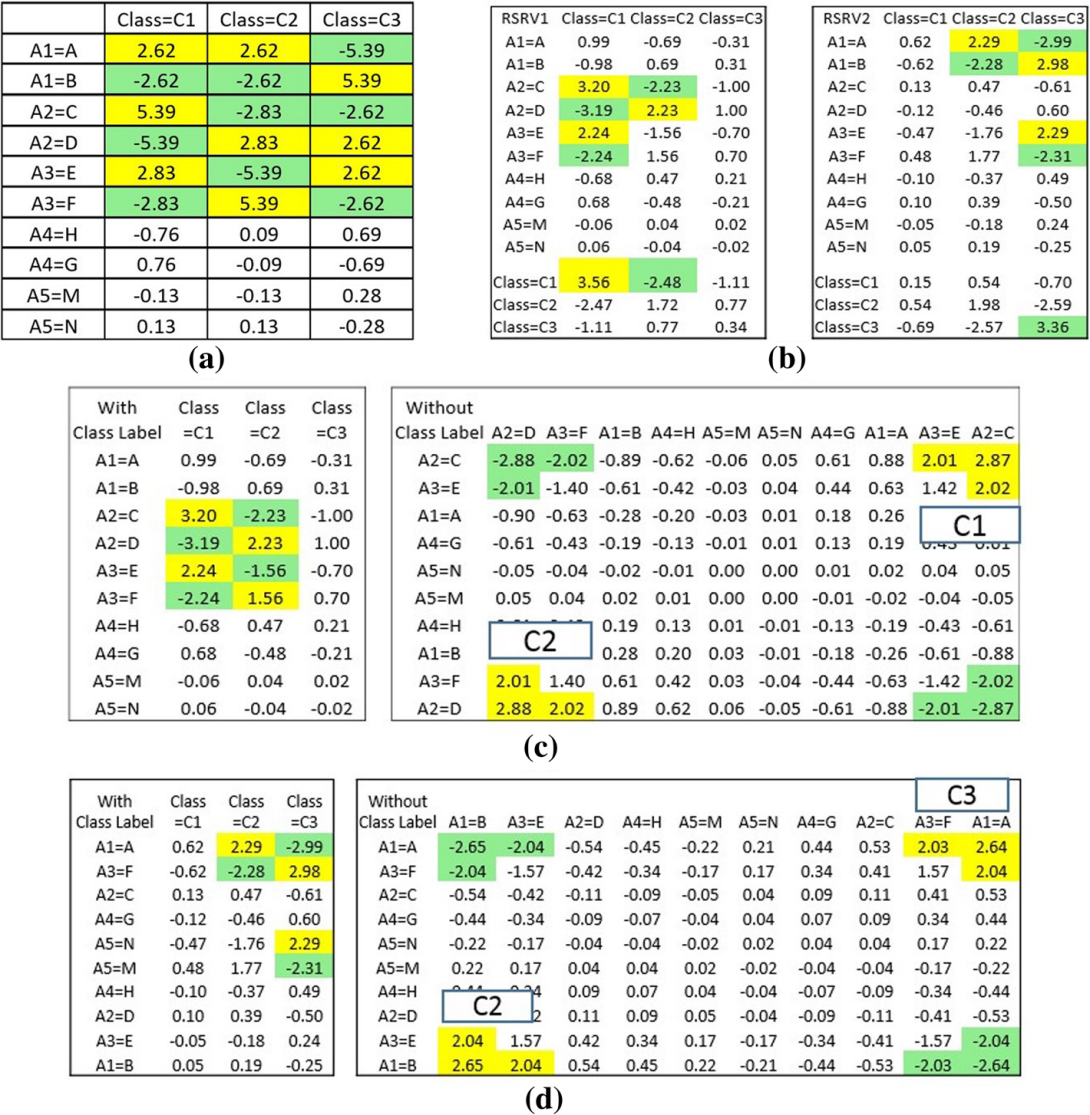
Pattern entanglement and disentanglement. ARA patterns are shown in significant SR colored in yellow. **a** A1A is entangled in class 1 and class 2; A2D is entangled in class 2 and class 3; and A3E is entangled in class 2 and class 3. **b** AR patterns disentangled in two RSRVs, pattern for classes 1 and 2 in RSRV1 and classes 2 and 3 in RSRV2. Note the different ARAs of class 2 --- one with the same residue site A2 as Class 1 but different ARs (A2C and A2D) while the other with site A1 with different ARs (A1A and A1B) with class 3. **c** and (**d**) show the two different sets of ARs, one associating with classes 1 and 2 and another with classes 2 and 3

Figure 5 show the result using adjusted residual as the measurement. In Fig. 5(a), we found that A1A is entangled in class 1 and class 2; A2D is entangled in class 2 and class 3; and A3E is entangled in class 2 and class 3. Later, after the disentanglement, the AVAs results are shown in RSRVs (Fig. 5(b)-(d)). We noted that the class patterns are disentangled. In Fig. 5(b), after disentanglement, RSRV1 captured the disentangled AVA patterns for class 1 and class 2. An interesting characteristic of this association is that they share the same AV in A2 but with different residues D and E. Their AV-vectors are on the opposite side of the same PC. RSRV2 reveals another set of associations between class 2 and class 3. Here they both involve A1 but with different values A and B. This shows that Class 2 has two association patterns, one associated with Class 1 and another associating with Class 3, just as what we implanted. They were disentangled in different PCs and RSRVs. Fig. 5(c) and (d) unveil all their disentangled patterns as implanted, with or without class labels given --- a robust demonstration of the deep knowledge discovered from the entangled source environment without the explicit reliance of prior knowledge or posteriori fixing.

### Bio-sequence dataset (cytochrome c protein family)

For protein study, we used three datasets. Dataset 1 and Dataset 2 are APCs obtained from two distinct localized regions from the dataset in [3] collected from the cytochrome c protein family with taxonomic class labels. In addition, Dataset 3 is the APC obtained from the class A scavenger receptors (SR-A) dataset in our previous paper [26] where we have reported some experimental result. In this paper we just highlight the use of address table in ARADD to track down the locations of ARAs we discovered and disentangled as detailed in [26].

*Dataset 1* is an APC dataset (width: 27) used in [3, 7] that contains 85 samples from four classes: Mammals, Plants, Fungus, and Insects. To compress this dataset, we reduced the number of aligned sites from 27 to 9 by removing the aligned sites with low SR2 value [13].

*Dataset 2* is an APC dataset (width:36) used in [3, 7] that contains 147 samples from six classes: Mammals, Birds, Fish, Insects, Metazoas and Plants. Like the dimensionality reduction process in Dataset 1, we reduced the dimensions from 36 to 17.

*Dataset 3* is an APC dataset (width: 12) used in [26] converting 95 protein sequences from five classes: Macro, Sra, Scara3, Scara4, Scara5 of class A scavenger receptors (SR-A) originally taken from a dataset with 106 sequences used in [27], one with the highest coverage. All five subclasses of proteins contain domains: Cytoplasmic, Collagenous, Transmembrane, a-helical and coiled-coil motifs. Macro, Sra, and Scara5 contain the Collagenous domain. Only Sra contains the SRCR domain.

In this paper, we first reported the results when E-ARADD was applied to the two datasets above, using both their APCs and APPCs. Analysis I focuses on evaluating and comparing the entangled ARAs and disentangled ARAs results for APCs composed of amino acid symbols for Dataset 1 and Dataset 2. Analysis II shows how E-ARADD being applied to the mixed-mode APPC for both datasets. Then, in the Discussion Section, we summarized the results of our work on dataset 3 reported in [26], highlighting how ARADD is able to reveal and locate all the significant ARs and ARAs inherent in an APC obtained from the sequence data of SR-A protein family. Since the AR and ARA ID Address Table reported in [26] is a special module of E-ARADD, we will include a brief summary the work in [26] in the discussion of this paper. We will briefly describe how E-ARADD is able to unveil the crucial functional information, of “what” and “where” of a protein family through the APCs discovered in the data [26].

### Analysis I – Cytochrome c APCs in amino acid symbols

**Fig. 6:**
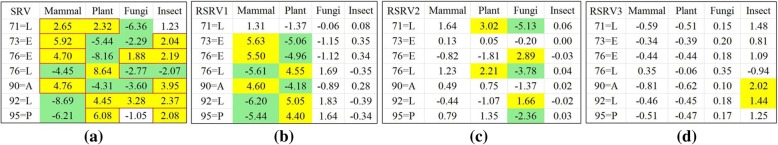
Discovered ARAs by E-ARADD for Dataset 1 with Amino Acid. (**a**) Entangled ARAs associating with classes in SRV; (**b**) Disentangled ARAs associating with Mammal and Plant in RSRV1; (**c**) Disentangled ARAs associating with Plant and Fungi in RSRV2; (**d**) Disentangled ARAs associating with Insect in RSRV3

**Fig. 7:**
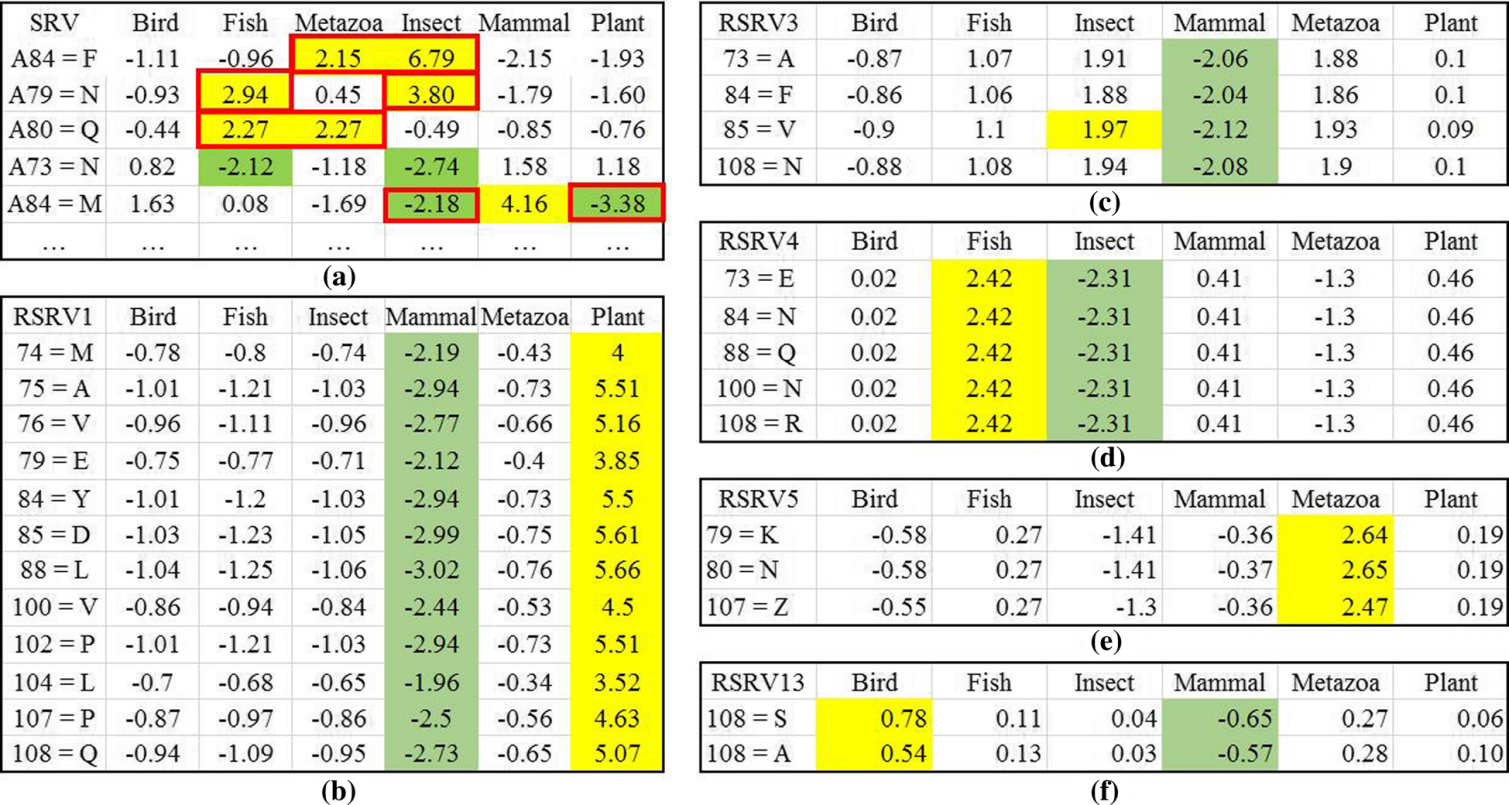
Discovered ARAs by E-ARADD for Dataset 2 with Amino Acid. (**a**) Entangled ARAs associating with classes in SRV; (**b-f**) Disentangled ARAs in RSRV1, RSRV3, RSRV4, RSRV5 and RSRV13

In Analysis I, we applied E-ARADD on APCs in amino acid symbols from data of dataset 1 and dataset 2. First, we compared the discovered ARAs obtained in RSRVs by using E-ARADD with those using only the adjusted statistical residual in SRV [28] with the same threshold 1.96. Figures 6 and 7 show the result of dataset 1 and dataset 2 respectively.

Figure 6(a) presents the results when the SRV was used to reveal the ARs associating with classes in dataset 1. From the SRV obtained from the APC, we observed that different species share the same ARs. For example, both Mammal and Plant share A71L. In another word, the ARs are entangled among different classes. However, after the E-ARADD disentanglement, we noted that the ARAs associating with class were disentangled as manifested in the RSRVs (Fig. 6b-d). In Fig. 6(b), ARs associating with Mammal were disentangled with those with Plant whereas most of them were quite mixed in the SRV (Fig. 6(a)). For instance, A92L was entangled among Plant, Fungi and Insect in SRV, but associates with Plant but not Mammal in the specific statistic/functional space RSRV1; and with Fungi in RSRV2 and Insect in RSRV3. This indicates that 192 L play different role in three uncorrelated statistic/functional spaces (though the latter could be weak, with SR = 2.02 and 1.44 respectively). We also observed that in RSRV3, only A90A and A92L associating with Insect were picked up. Note that the weak association of A92L with Insect (SR = 1.44) will play a strong role (SR = 5.05) in Plant in RSRV1 and a weaker role (SR = 1.66) in RSRV2. The importance of E-ARADD Disentanglement of ARAs with different classes were clearly revealed in different statistical/functional spaces, RSRV1, RSRV2 and RSRV3, as captured through their corresponding PCs.

Similarly, Fig. 7 shows the discovered ARAs on SRV and RSRVs from the APC in amino acid symbols from dataset 2. Figure 7(a) shows the result in SRV. Here, we observed that “Mammal” stands out with positive SR associating with A84M while other ARs were entangled with different classes. Note that from the SRV obtained from this APC, Birds and even Plants were irrelevant. We also noted that ARs associating with Metazoa, Insect and Fish were mixed. However, after the disentanglement, the result of RSRVs shown in Fig. 7(b-f) told a different story. In Fig. 7(d) Fish stands out from Insect and Metazoa. In Fig. 7(e) Metazoa separates from Insect and Fish. The ARA with specific classes stood out in different disentangled spaces. More surprising is that the AR missing in the Bird class appeared in *PC*_13_ and *RSRV*_*13*_ with low SR but its ARA values still stand out from the SRs of all the other ARAs. This indicates the capability of ARADD in revealing weak ARAs (rare events) encountered in the imbalanced class problem that has plagued data mining for sometimes [29].

This experimental result shows that, beside discovering and disentangling the ARAs, E-ARADD can discover AR Clusters (ARCs) and significant ARs captured in orthogonal PCs and their corresponding RSRVs. It demonstrated the Explainable AI (XAI) [36] capability without the reliance of explicit a priori knowledge and a posteriori processing.

**Fig. 8:**
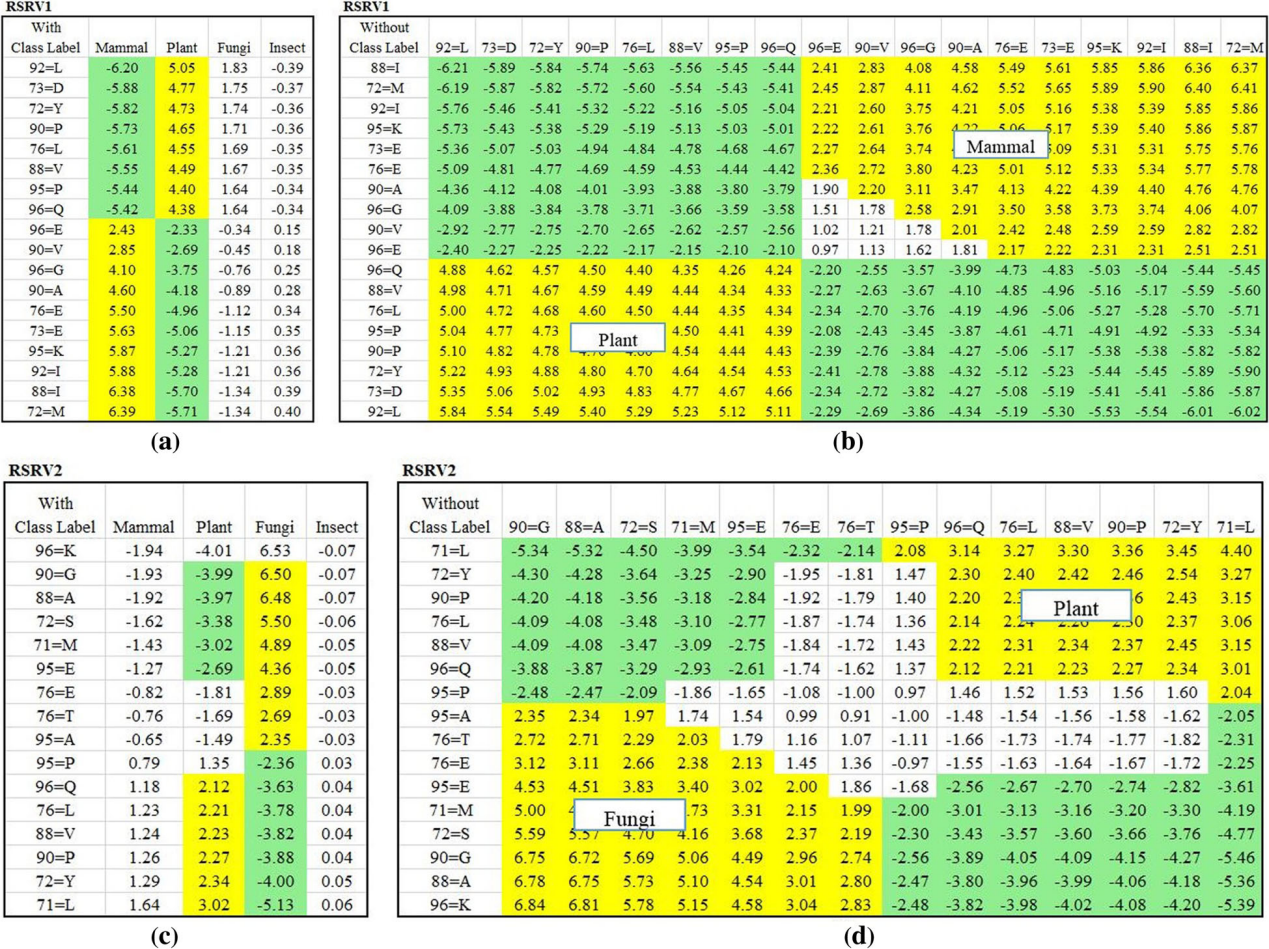
The Result of AR Clusters captured in PCs and the corresponding ARAs reflected in RSRVs for APC Dataset 1 with amino acid. **a** ARA Clustering Result with Class Label on RSRV1; (**b**) ARA Clustering Result without Class Labels on RSRV1 (**c**) ARA Clustering Result with Class Label on RSRV2; and (**d**) ARA Clustering Result without Class Labels on RSRV2

**Fig. 9:**
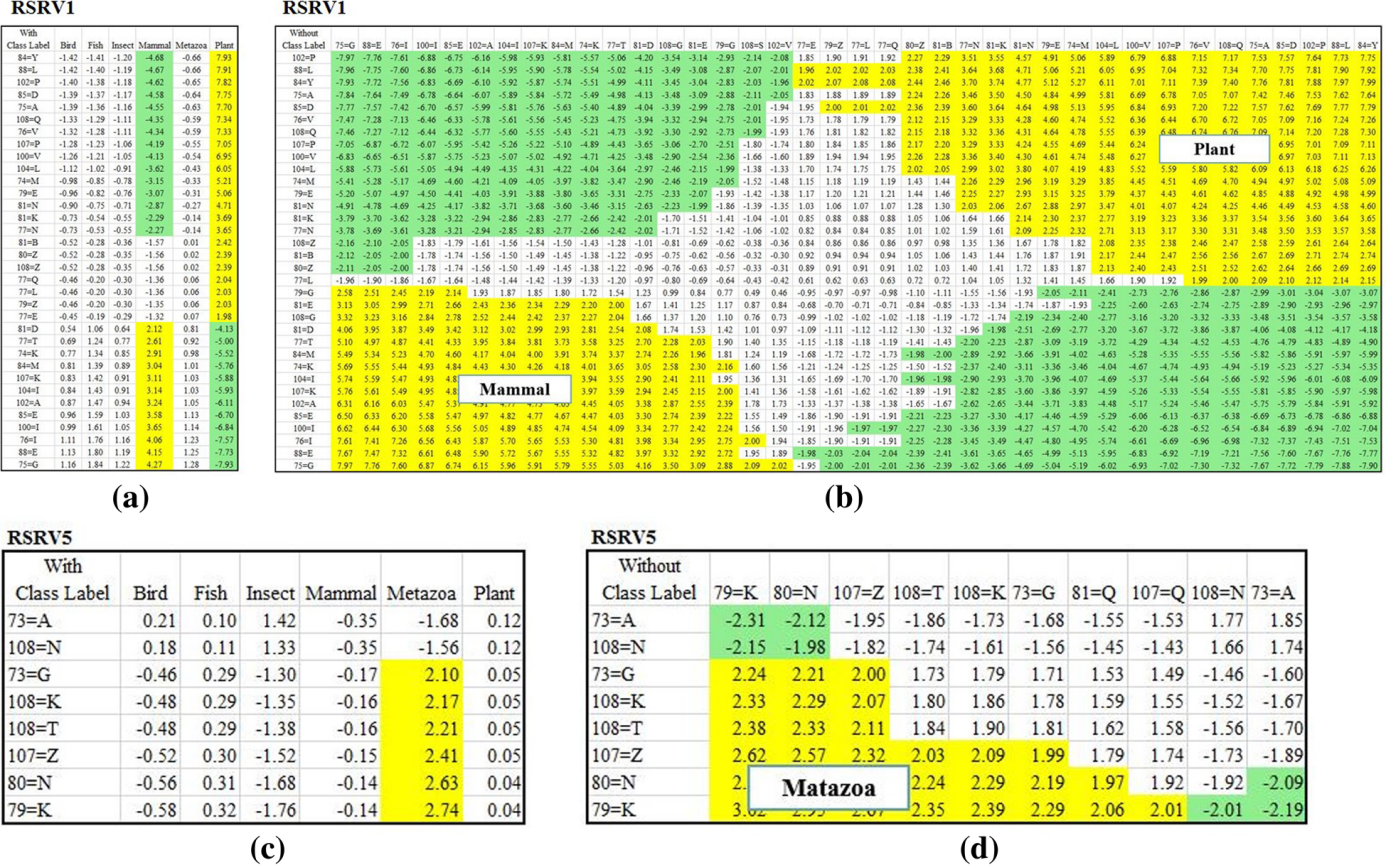
The Result of AR Clusters captured in PCs and the corresponding ARAs reflected in RSRVs for APC Dataset 2 with amino acid. **a** ARA Clustering Result with Class Label on RSRV1; (**b**) ARA Clustering Result without Class Labels on RSRV1; (**c**) ARA Clustering Result with Class Label on RSRV5; and (**d**) ARA Clustering Result without Class Labels on RSRV5

To reveal the ARAs obtained for dataset 1 in greater depth, we made a careful comparison of the ARs in the PCs with the ARAs in their corresponding RSRVs to see how the ARAs grouping reflecting the distinctness of the AR sub-clusters in the RSRVs. Fig. 8 shows the AR clusters (yellow cells) that captured on different PCs. In order to show that such functional associations are intrinsic unrelated to class labels, we compared experimental result on APCs with and without class labels. Figure 8 (a-d) showed the AR clusters on the right-handed side and their corresponding RSRV plots obtained from SRV without class labels on the left-handed side. We observed in both that the ARs associating to the class are almost identical. Hence, this further indicates the explainable machine learning capability of E-ARADD in both supervised/unsupervised settings not relying on explicit a priori or a posteriori knowledge. As Figs. 8 and 9 show, in all the experiments, we see little difference in ARA results with or without class labels given.

### Analysis II – Cytochrome c APCs in aligned residue property tuples

In Analysis II, the same protein APC datasets in Analysis I were used, but the aligned residues are represented by the five amino acid chemical properties: Side Chain Polarity, Side Chain Acidity / Basicity, Hydropathy Index, Molecular Weight (Da), and Isoelectric Point instead. Thus, we represent an APC in Analysis I by an Aligned Property Pattern Cluster (APPC) and an Aligned Residue (AR) by an Aligned Residue Property Tuple (ARP). Furthermore, instead of using ARAs as our fundamental association from the APC from Dataset 1, we used the Aligned Residue Property Association (ARPA) obtained from APPCs instead.

**Fig. 10:**
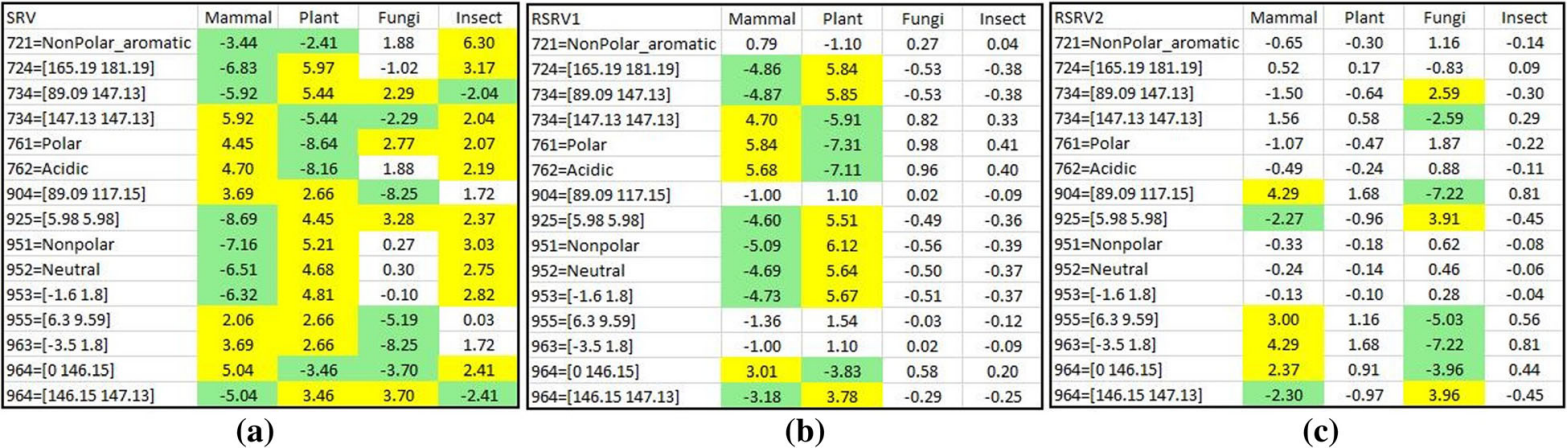
Discovered ARPAs on SRV and RSRVs by E-ARADD for APC Dataset 1 with chemical properties. **a** Entangled ARPAs with class on SRV; (**b**) Disentangled ARPAs on RSRV1; (**c**) Disentangled ARPAs on RSRV2

In this paper, our focus is not to conduct a thorough bio-molecular study of a protein family but rather to explore the performance of E-ARADD on APPCs. That we selected the APCs from cytochrome C based on amino acids and their chemical properties is to examine whether ARPAs can be discovered in the disentangled spaces of PCs and RSRVs to reveal the chemical association ARPs at a deeper level. We would also like to find out also whether ARP clusters could be identified to generate ARP subgroups corresponding to taxonomical classes with or without class label provided. Thus, we took the dataset 1 and converted the APC with a width of 9 amino acids into a 9×5 mixed-mode APPC, from which we constructed an SRV. We then applied PCD on the SRV to obtain PCs and RSRVs as we did in Analysis I, ranked them after their eigenvalues. The corresponding set of RSRVs then represent the coordinates of the ARP-vector which were the SRs of each ARPA between ARPs corresponding to the row and column ARP-vectors. Figure 10 shows the disentanglement of the ARPs associating with class labels for dataset 1. The attribute “721 = NonPolar_aromatic” denotes that the aligned 1st chemical property (Side Chain Polarity) of the 72th amino acid in the APPC is “NonPolar_aromatic”. Since chemical properties were used, we observed more disentangled association of the ARP with class labels were obtained in the SRV (Fig. 10(a)). As expected, more succinct disentanglement had also been observed in the RSRVs. In RSRV1 (Fig. 10(b)), we observed succinct disentanglement of ARPAs between Mammal and Plant, and in RSRV2 (Fig. 10(c)), between Mammal and Fungi. Overall, we see that more specific chemical associations between species are discovered in different functional spaces. Such deeper knowledge could help biologists to further their research.

**Fig. 11:**
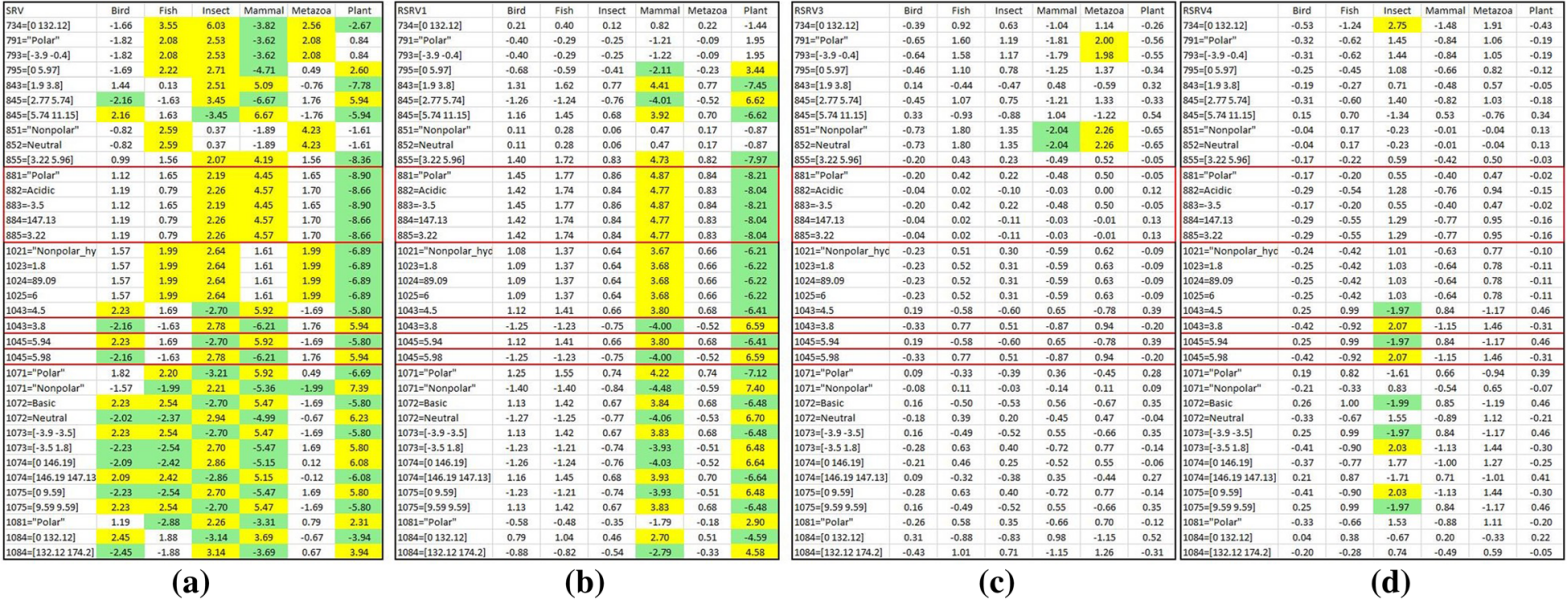
Discovered ARAPs on SRV and RSRVs by E-ARADD for APC Dataset 2 with chemical properties. **a** Entangled ARPAs with class on SRV; (**b**) Disentangled ARPAs on RSRV1; (**c**) Disentangled ARPAs on RSRV3. **d** Disentangled ARPAs on RSRV4

Similarly, Fig. 11 shows the ARPAs with the class labels in disentangled spaces for dataset 2. Figure 11(a) shows the result of ARP Associating with classes in the SRV obtained from the APPC with chemical properties of dataset 2. Note that in SRV, we noted that there are quite a number of ARP entangled with different taxonomical classes. However, after disentanglement, we observed that disentangled ARPs in RSRV1 were distinctly associating Mammal and Plant. Especially for site *1043 = 3.8* (the value of the 3th properties of the 104th amino acid), and *1045 = 5.98*, While ARPs of Insect and Plant are entangled in SRV, the ARP of Insect was standing out in RSRV3 and that of Plant was standing out in RSRV1.

**Fig. 12:**
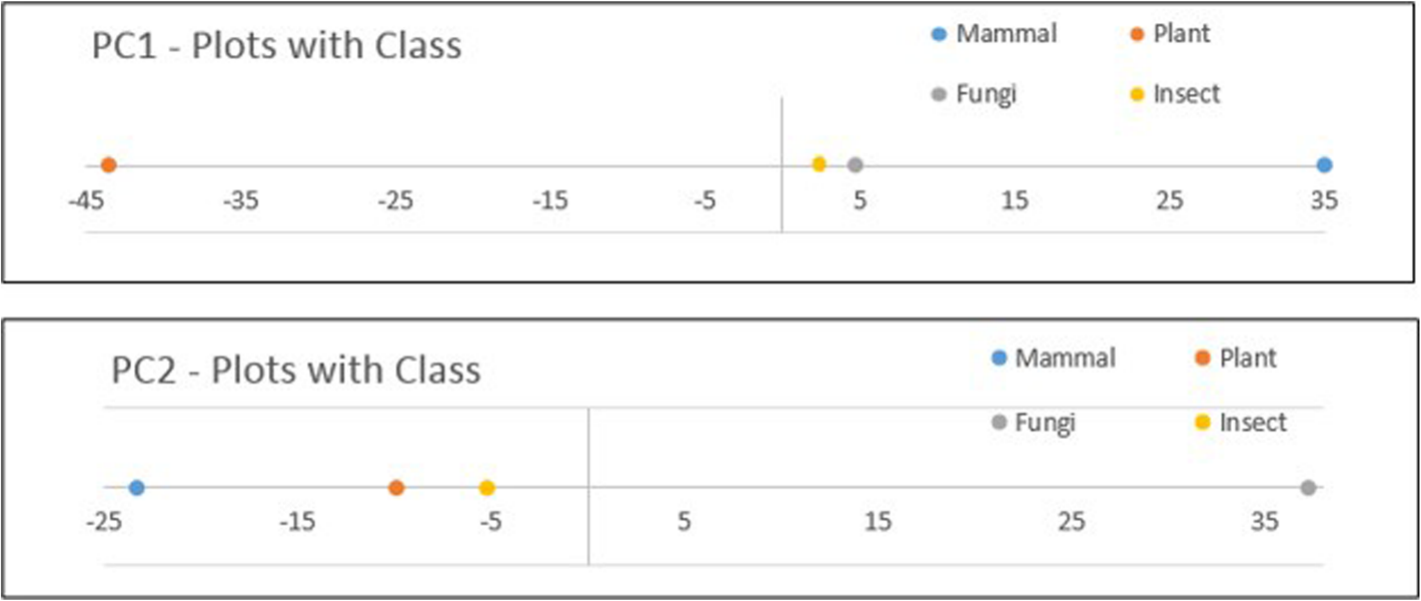
PC plots results for APPC in dataset 1 with class label

**Fig. 13:**
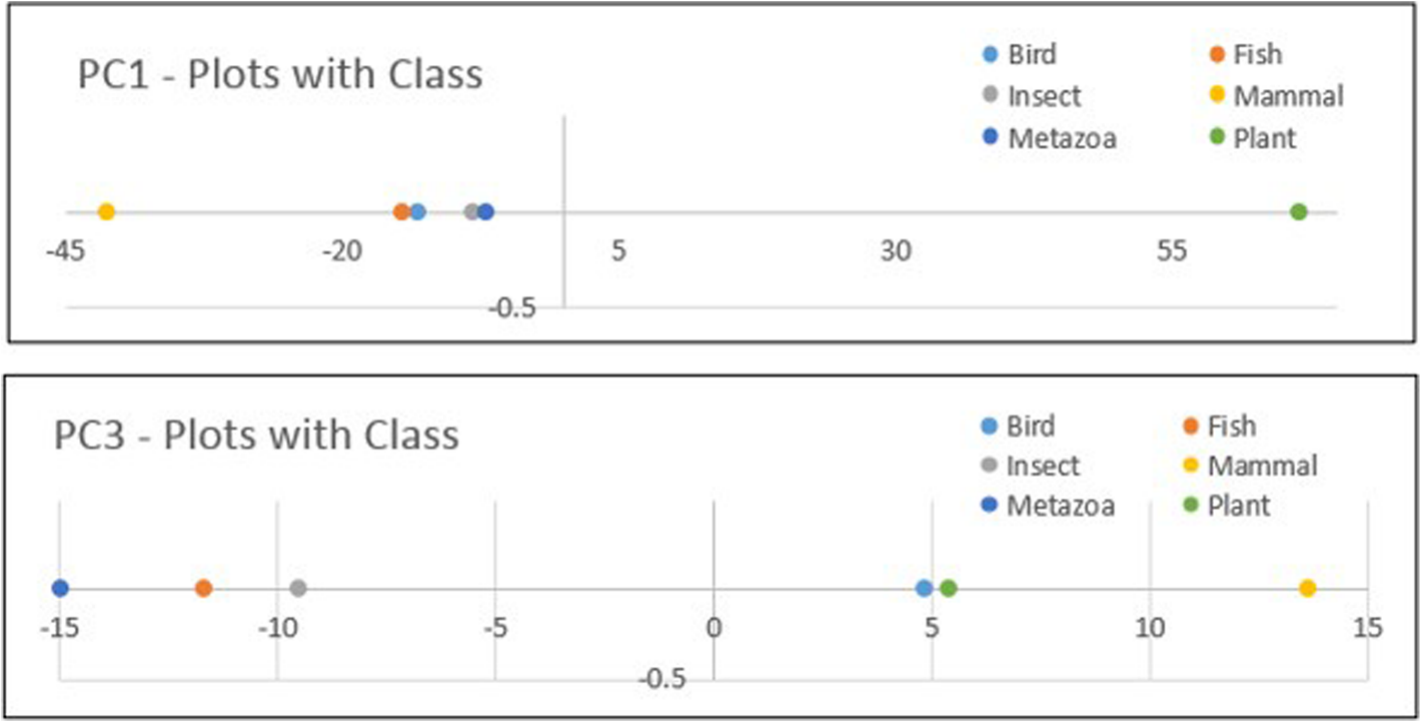
PC plots results for APPC in dataset 2 with class label

We can conclude from the above experimental result that when the class labels are included in APPCs as input, we could disentangle the discovered associations of chemical property relation between ARPs. In addition, we also showed from the PC plots for dataset 1 and 2 how chemical properties of the ARP clusters were associating with class in the PCs as Figs. 12 and 13 show. Since each ARP-cluster consists of a special set of ARPs, biologists could gain significant molecular biological insight for each specific functional space. Such ARP associating with classes were also revealed in other RSRVs.

In summary, from the results of Analysis II, we found that E-ARADD can handle mixed-mode dataset. It can discover the statistically significant ARPAs, though entangled in the SRV, as well as the ARP Clusters (ARPCs) captured in orthogonal PCs to bring out their separability associating with taxonomical classes.

## Discussion

Discovering patterns from biological sequences is of fundamental importance in unraveling the underlying science. It is particularly true in Proteomics, where proteins virtually regulate every biological process of a living organism. A new method has been developed from us to obtain from protein sequences Aligned Pattern Clusters (APCs) [2–4] representing the biological conserved regions. APCs [2–4], comparing with probabilistic methods [1], have captured more complete statistical association of aligned residues. As the column-wise associations are preserved in APCs, contrasting to probabilistic models, e.g. Position Weight Matrix (PWM) [30], we are able to discover the Aligned Residue Associations (ARAs) [5] to reveal subgroup characteristics, in which, these subgroup characteristics, regarding multiple functionalities and/or local stereo physiochemical environments, may have been masked or entangled. We further extended the ARADD algorithm into E-ARADD to handle the mixed-mode biochemical protein data to provide direct biochemical interpretation supported by experimental results.

Experimental results on synthetic data provides the proof-of-concept validation on the successful disentanglement that reveals class-associated ARAs with or without class labels as input, results on cytochrome c and class A scavenger receptors sequence data render scientific validation of our method. In experimental Analysis I, after disentanglement, different ARAs were revealed. They were linked to the species class labels. We validated that the AR results in PCs and ARAS in RSRVs remain essentially the same with or without the inclusion of class labels in the APCs. In experimental Analysis II, we found that different APPAs were associated with different species. The observation, to a certain degree, is consistent to the literature report that certain biological processes of cytochrome c such as oxidization have homologous yet different chemical characterization in different families [31].

Furthermore, in order to show the completeness of the proposed algorithm E-ARADD, we furnish a brief summary of our recent work [26] when ARADD algorithm [5] was applied to a very diverse protein family of class A scavenger receptors (SR-A), dataset 3. In our recent work [26], we showed that ARADD was able not only to discover and disentangle ARs and ARAs in specific PCs and RSRVs, but also their locations in the protein functional domains of SR-A.

**Fig. 14:**
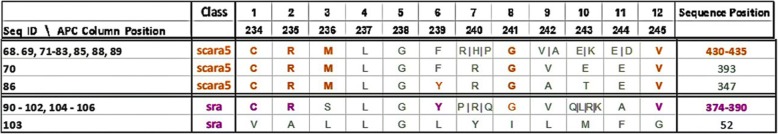
An Excerpt of Experimental result of AR groups associating with different SR-A classes [28]

Figure 14 demonstrates an excerpt showing the results of only two classes from a figure taken from our recent work [26]. Note that the AR patterns are in bold brown color fonts for Scara5 (CRM****G***V) and in violet color fonts for Sra (CR***Y*G***V). These AR patterns are similar in sequence and thus clustered in the same APC. However, they are in fact in two distant domains. This indicates that not only can ARADD disentangle functional association in an APC, i.e. the pattern space, but also disentangle their sequence locations relating to different family domains [26], e.g. Scara5 and Sra. This provides a strong support to the scientific significance of ARA disentanglement, by revealing the information of “what” and “where” in a protein family.

**Fig. 15:**
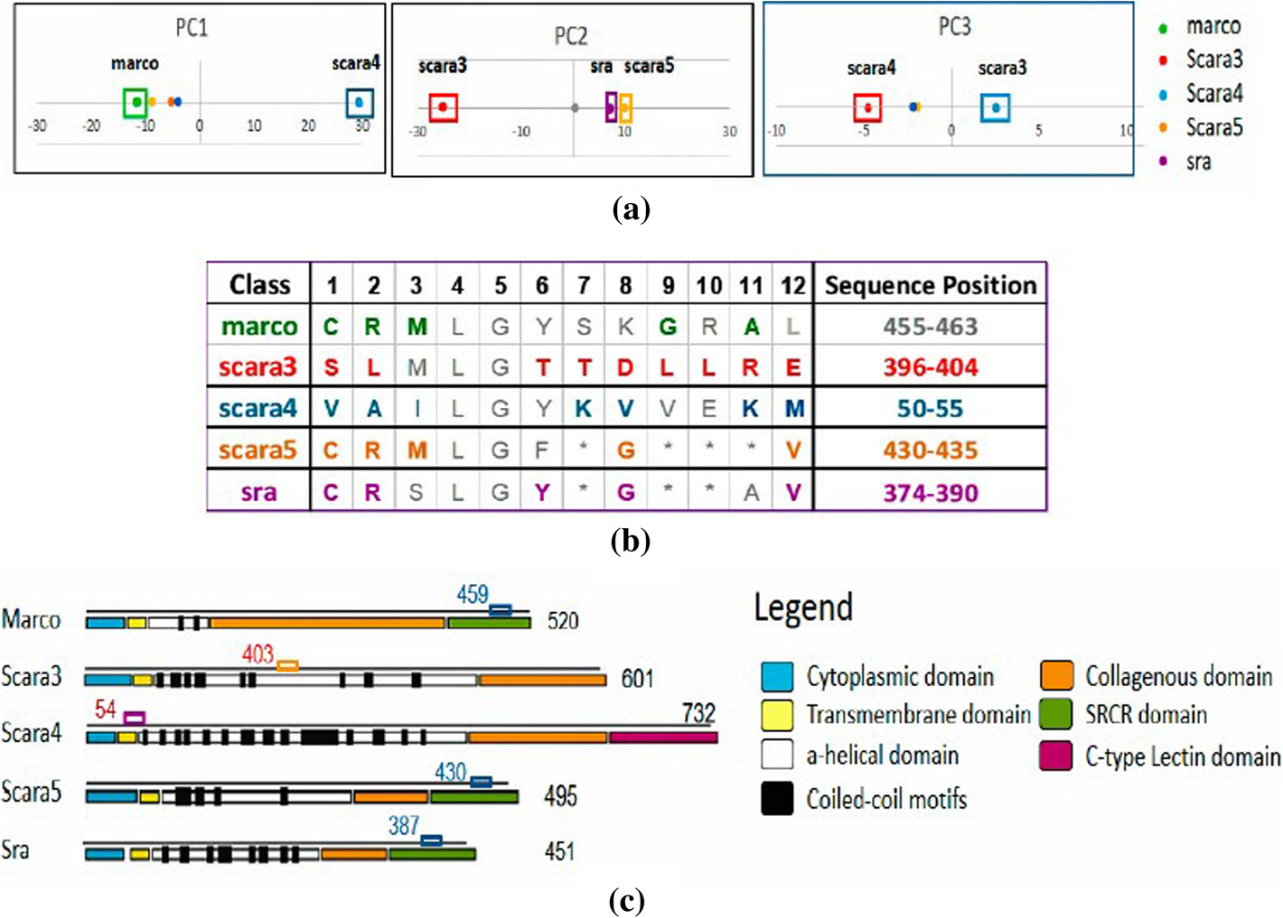
The process of Aligned Residue Association (ARA) disentanglement in both pattern space and sequence location. **a** the result of disentanglement of functional groups corresponding to classes; (**b**) an APC with AR groups in different bold colors corresponding to different classes with the range of positions shown; (**c**) the 5 AR groups mapped onto the protein sequences with domain regions annotated [28] and class labels associated [26]

Figure 15 [26] provides an overview of the discovered results in both pattern and data space. Figure 15(a) from [26] demonstrates that the class labels associating with ARs of their pertaining classes are revealed within their associating clusters in the one-dimensional PC space. As shown in Fig. 14, the AR groups for Scara5 and Sra are close with only a single difference in their significant ARs. Their closeness is also observed in RSRV2 [26]. The two groups differ from other classes significantly. Hence, from the PCs (Fig. 15(a)) and the plots of the significant AR clusters (color rectangular boxes), we have observed both their similarity (i.e. Sra and Scara5 in PC2) and their differences (i.e. scara3 and scara4 in PC3), with statistical backing (the distance of their projection position from the mean in the PCs and their SR magnitude in the RSRVs). From the APC data space as shown by their sequence ID and sequence position in Figs.14(a), 15(b) and (c), we observed that their residing sequence positions and family domain locations of each AR pattern were identified. Surprisingly, they are closely correlated with the domain regions annotated.

## Conclusion

In this study, we extend our previous ARADD algorithm [5] into E-ARADD to enable it to handle mixed-mode physiochemical property data, which contains both categorical and numerical values. By applying E-ARADD to the entangled APC obtained from cytochrome c family and class A scavenger receptors, this study has shown that AR clusters (patterns in pattern space), associating with different functional subgroups, regions and domains of the family obtained from an APC, could be succinctly plotted and statistically separated in different PCs and RSRVs as well as in different locations through their sequence ID and sequence position in the protein family data [26].

The most significant finding of this study is that the AR subgroups within the APCs could be found in the disentangled PCs and RSRVS of ARA/ARPA associating with different classes or subgroups, residing in different functional regions or domains of the family. Biologically, entangled ARA/ARPA in the aligned patterns within the conserved regions APC/APPC of class A scavenger receptor, reveal biological functional patterns pertaining to similar or different classes. It is interesting to find that the ARAs/ARPAs within the entangled patterns in APCs of class A scavenger receptor family can be disentangled into subgroups pertaining to different functionality as reflected by the disentangled PCs and RSRVs. This implies that the strong statistical associations of multiple functionalities for different classes/subgroups inherent in the residue associations within the aligned patterns. Hence, in summary, the successful application of ARADD algorithm demonstrates its capability to open a new way for analyzing conserved regions and their distribution, with potential to reveal new knowledge in omics for drug discovery, genetic medicine and gene therapy applications.

## Abbreviations

APC: Aligned Pattern Cluster (with categorical amino acid)
APPC: Aligned Property Pattern Cluster (with mixed-mode chemical properties)
AR: Aligned Residue (for categorical amino acid in APC dataset)
ARA: Aligned Residue Association (Significant co-occurrence of two ARs in APC / APC of biochemical properties)
ARA/ARPA FM: ARA/ARPA Frequency Matrix
ARP: Aligned Residue Property (for mixed-mode chemical properties in APC dataset)
ARPA: Aligned Residue Property Association (for APPC)
PCD: Principle Component Decomposition
RSRV: Re-projected SRV
SR: adjusted Statistical Residual between two ARs/ARPs
SRV: ARA/ARPA adjusted Statistical Residual Vector Space

## References

[1] Durbin R, Eddy S, Krogh A, Mitchison G. Biological sequence analysis: Probabilistic Models of Proteins and Nucleic Acids. Analysis. 1998;356 Available from: https://pdfs.semanticscholar.org/2ed5/d6b35f8971fb9d7434a2683922c3bfcc058e.pdf.

[2] Lee En-Shiun, Wong Andrew KC (2013). Ranking and compacting binding segments of protein families using aligned pattern clusters. Proteome Science.

[3] Wong AKC, Lee ESA (2014). Aligning and clustering patterns to reveal the protein functionality of sequences. IEEE/ACM Trans Comput Biol Bioinforma.

[4] Sze-To A, Wong AKC. Pattern-Directed Aligned Pattern Clustering. Bioinforma. Biomed. (BIBM), 2017 IEEE Int Conf IEEE; 2017.

[5] Zhou P, Sze-Tzo A, Wong AKC. Discovery and disentanglement of protein aligned pattern clusters to reveal subtle functional subgroups, 2017. Kansas: IEEE International Conference on Bioinformatics and Biomedicine (BIBM), MO. 2017; pp. 62–69. http://ieeexplore.ieee.org/stamp/stamp.jsp?tp=&arnumber=8217625&isnumber=8217602.

[6] Wong AKC, Zhou P, Sze-To A. Discovering Deep Knowledge from Relational Data by Attribute-Value Association. In: Proceedings of the 13th International Conference on Data Mining (DMIN’17), Las Vegas, NV, USA. 2017. p. 51–57. https://csce.ucmss.com/cr/books/2017/LFS/CSREA2017/DMI8008.pdf.

[7] Zhou P, Lee E-SA, Wong AKC. Regrouping of pattern clusters to reveal characteristics of distinct classes and related classes. Proc. - 2013 IEEE Int. Conf. Bioinforma. Biomed. IEEE BIBM 2013. 55–61.

[8] Naulaerts S, Meysman P, Bittremieux W, Vu TN, Vanden BW, Goethals B (2015). A primer to frequent itemset mining for bioinformatics. Brief Bioinform.

[9] Agrawal R, Imielinski T, Swami A. Mining Association in Large Databases. Proc 1993 ACM SIGMOD Int Conf Manag data - SIGMOD ‘93. 1993:207–16.

[10] Han Jiawei, Pei Jian, Yin Yiwen, Mao Runying (2004). Mining Frequent Patterns without Candidate Generation: A Frequent-Pattern Tree Approach. Data Mining and Knowledge Discovery.

[11] Edgar Robert C, Batzoglou Serafim (2006). Multiple sequence alignment. Current Opinion in Structural Biology.

[12] Notredame C (2007). Recent evolutions of multiple sequence alignment algorithms. PLoS Comput Biol.

[13] Thompson Julie D., Linard Benjamin, Lecompte Odile, Poch Olivier (2011). A Comprehensive Benchmark Study of Multiple Sequence Alignment Methods: Current Challenges and Future Perspectives. PLoS ONE.

[14] Frith MC, Hansen U, Spouge JL, Weng Z (2004). Finding functional sequence elements by multiple local alignment. Nucleic Acids Res.

[15] Bailey TL, Elkan C (1995). Unsupervised learning of multiple motifs in biopolymers using expectation maximization. Mach Learn.

[16] Altschuh D, Lesk AM, Bloomer AC, Klug A (1987). Correlation of co-ordinated amino acid substitutions with function in viruses related to tobacco mosaic virus. J Mol Biol.

[17] Kass Itamar, Horovitz Amnon (2002). Mapping pathways of allosteric communication in GroEL by analysis of correlated mutations. Proteins: Structure, Function, and Genetics.

[18] Zani Izma, Stephen Sam, Mughal Nadeem, Russell David, Homer-Vanniasinkam Shervanthi, Wheatcroft Stephen, Ponnambalam Sreenivasan (2015). Scavenger Receptor Structure and Function in Health and Disease. Cells.

[19] Ma PCH, Chan KCC (2011). Incremental fuzzy mining of gene expression data for gene function prediction. IEEE Trans Biomed Eng.

[20] Jiawei H, Kamber M, Han J, Kamber M, Pei J. Data Mining: Concepts and Techniques [Internet]. San Fr. CA, itd Morgan Kaufmann. 2012. Available from: http://scholar.google.com/scholar?hl=en&btnG=Search&q=intitle:Data+Mining+Concepts+and+Techniques#1%5Cn, http://scholar.google.com/scholar?hl=en&btnG=Search&q=intitle:Data+mining+concepts+and+techniques%231%5Cn, http://scholar.google.com/scholar?hl=en&btnG=Se.

[21] Ramoni M, Sebastiani P, Cohen P. Multivariate clustering by dynamics Marco. Drugs. 2001:1–68.

[22] Wong Andrew K. C., Wang David C. C. (1979). DECA: A Discrete-Valued Data Clustering Algorithm. IEEE Transactions on Pattern Analysis and Machine Intelligence.

[23] Liu L, Wong AKC, Wang Y (2004). A global optimal algorithm for class-dependent discretization of continuous data. Intell Data Anal.

[24] Wong AK, Wu B, Wu GP, Chan KC. Pattern discovery for large mixed-mode database. Proc 19th ACM Int Conf Inf Knowl Manag. 2010:859–68.

[25] Shlens J. A tutorial on principal component analysis. ArXiv. 2014:1–13. https://arxiv.org/pdf/1404.1100.pdf.

[26] Zhou Pei-Yuan, Lee En-Shiun, Sze-To Antonio, Wong Andrew (2018). Revealing Subtle Functional Subgroups in Class A Scavenger Receptors by Pattern Discovery and Disentanglement of Aligned Pattern Clusters. Proteomes.

[27] Lee E-SA, Whelan FJ, Bowdish DME, Wong AKC (2016). Partitioning and correlating subgroup characteristics from aligned pattern clusters. Bioinform.

[28] Whelan Fiona J, Meehan Conor J, Golding G Brian, McConkey Brendan J, E Bowdish Dawn M (2012). The evolution of the class A scavenger receptors. BMC Evolutionary Biology.

[29] Sun Y, Kamel MS, Andrew KCW, Wang Y (2007). Cost-sensitive boosting for classification of imbalanced data. Pattern Recogn.

[30] Xia X. Position weight matrix, Gibbs sampler, and the associated significance tests in motif characterization and prediction. Scientifica (Cairo). 2012;2012. 10.6064/2012/917540 PMC3820676 24278755

[31] Popovic DM, Leontyev IV, Beech DG, Stuchebrukhov AA (2010). Similarity of cytochrome c oxidases in different organisms. Proteins Struct. Funct. Bioinforma.

